# Supplementary material for: Blood Bacterial DNA Load and Profiling Differ in Colorectal Cancer Patients Compared to Tumor-Free Controls
Source: Cancers (Basel). 2021 Dec 18;13(24):6363. doi: 10.3390/cancers13246363 (PMC8699505; doi:10.3390/cancers13246363)

cancers

MDPI

Supplementary Material

Supplementary Methods

Bacterial DNA contamination assessment

To assess the potential bacterial DNA contamination from environment and reagents, several negative controls were included and analysed throughout the 16S rRNA gene sequencing pipeline. Ten negative controls consisting of ultrapure water (NC-EXT-WA) were added at the DNA extraction step to account for reagent and environmental contaminants. NC-EXT-WA samples were processed concomitantly with the DNA extraction from whole blood samples. This study comprised 296 whole blood samples. 16 rounds of DNA extraction were performed adding 2 NC-EXT-WA samples to the extraction round 1 (EXT1), 5 (EXT5), 10 (EXT10), 15 (EXT15) and 16 (EXT16).

To overcome the unbalance between the whole blood sample and the control groups (296 vs 10), we performed 10 randomized statistical analyses by comparing the 10 NC-EXT-WA samples to 10 randomly selected whole blood samples. The beta-diversity was measured through Bray-Curtis index. PERMANOVA and PERMDISP were used to test for homogeneity of multivariate dispersions on Bray-Curtis dissimilarity table for the contamination control.

We used diversity analyses and comparisons of the taxonomic profiles in order to confirm that the differences between DNA extraction negative controls and whole blood samples were large enough.

Beta-diversity indices were used to investigate whether blood bacterial DNA differs from that of extraction negative controls. Based on all beta-diversity indices, especially on the Bray-Curtis distance matrix (Supplementary Figure S4), blood bacterial DNA profiles were separated from the negative controls in all the randomized analyses. Moreover, blood samples tended to form a tighter cluster than the negative controls, suggesting that the blood samples shared a specific and more similar DNA bacterial profile. The negative control group was more dispersed, showing more variability in-between samples and suggesting a random profile more evident. PERMANOVA showed statistically significant differences between whole blood samples and negative controls for Bray-Curtis beta-diversity indices (Supplementary Table S2), suggesting that the bacterial communities differed both in composition and relative abundance between biological and negative control samples. PERMDISP confirmed the results.

We could, therefore, consider that the background noise did not impact the results of the study.

Table S1. Distributions of Observed, Chao1, Shannon and Simpson alpha-diversity indices of control, intestinal adenoma (IA), colon and rectal cancer subjects for bacterial genera and OTUs.

		**Median (I-III quartiles)**									
	Alpha-diversity	**Controls**	**IA**	**Colon cancer**	**Rectal cancer**	**p* Controls vs IA**	**p* Colon cancer vs IA**	**p* Colon cancer vs controls**	**p* Rectal cancer vs IA**	**p* Rectal cancer vs controls**	**p* Colon cancer vs rectal cancer**
**Genera**	*Observed*	28.5 (25–32)	28 (25–32)	29.5 (25–36)	30 (26–32)	0.917	0.246	0.251	0.364	0.332	0691
	Chao1	44.9 (33–57.5)	44.5 (34–55.5)	44.5 (36.3–55.3)	41.3 (35–53)	0.686	0.874	0.774	0.617	0.879	0.493
	Shannon	2.34 (2.02–2.53)	2.33 (2.15–2.51)	2.33 (2.00–2.58)	2.32 (2.12–2.51)	0.575	0.824	0.706	0.958	0.540	0.723
	Simpson	0.87 (0.80–0.89)	0.86 (0.83–0.89)	0.86 (0.81–0.90)	0.86 (0.82–0.89)	0.843	0.808	0.884	0.855	0.792	0.981
**OTUs**	*Observed*	34 (31–39)	35 (31–42)	37 (32–47)	37 (33–40)	0.542	0.208	0.105	0.440	0.171	0.617

Chao1	62.1 (48–85)	66 (49.2–90.5)	67.1 (51.0–87.5)	59.9 (49.6–82)	0.297	0.631	0.594	0.299	0.968	0.702
Shannon	2.58 (2.28–2.77)	2.58 (2.40–2.80)	2.61 (2.27–2.87)	2.51 (2.41–2.74)	0.372	0.874	0.496	0.625	0.667	0.581
Simpson	0.90 (0.86–0.92)	0.90 (0.87–0.92)	0.90 (0.86–0.92)	0.89 (0.88–0.91)	0.671	0.879	0.749	0.241	0.482	0.378

* P for heterogeneity estimated from the Wilcoxon rank-sum. SD: standard deviation; IQ: interquartile.

Table S2. PERMANOVA and PERMDISP on beta-diversity Bray-Curtis index for the 10 randomized comparisons between negative controls (CTRL) and whole blood samples (WB). For all randomized comparisons, the sample size is equal to 20 (10 Neg CTRL vs. 10 WB) at the exception of RandExt16 (10 Neg CTRL vs. 9 WB). The statistical testing used were pseudo-F and F-value for PERMANOVA and PERMDISP, respectively. For both tests, 2000 permutations were performed. P-values<0.05 in yellow, <0.005 in orange and <0.0005 in red.

			RandExt01	RandExt05	RandExt10	RandExt15	RandExt16	RandAllExt	Rand1	Rand2	Rand3	Rand4
Bray-Curtis	PERMANOVA	Test statistic	3,49	4,51	3,51	4,01	4,37	3,03	4,08	3,22	2,94	2,82
		P-value	0,0005	0,0005	0,0005	0,0005	0,0005	0,0005	0,0005	0,0005	0,0005	0,0005
	PERMDISP	Test statistic	10,67	29,79	15,52	13,87	28,65	9,96	15,13	18,59	8,28	3,60
		P-value	0,0050	0,0005	0,0025	0,0005	0,0005	0,0130	0,0005	0,0015	0,0190	0,0900

Figure S1. Distribution of 16S rRNA gene copies per μl of whole blood among controls, intestinal adenomas and colorectal cancers.

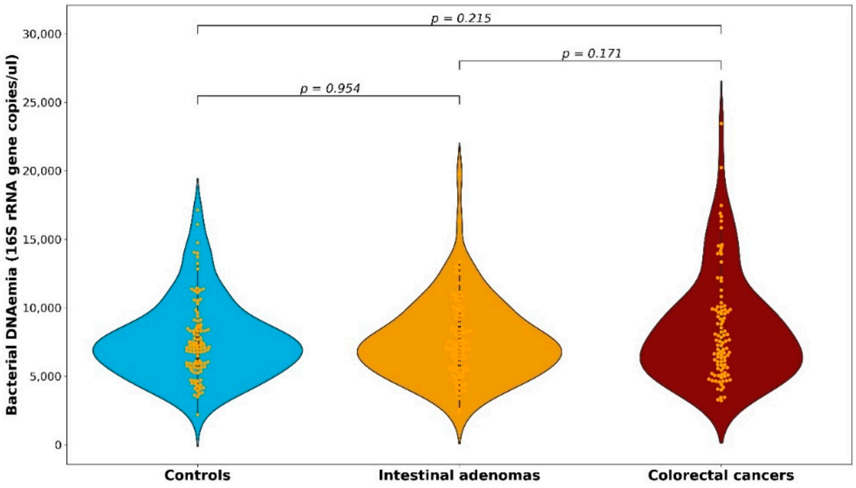

Figure S2. Mean of the taxonomy composition in terms of relative abundance per group of samples at family taxonomic level. The panel on the left shows the taxonomic composition of controls, intestinal adenoma subjects (IA) and both controls/IA, respectively (as per legend to the right side). The panel on the right shows the taxonomic composition of colorectal cancer (CRC), and rectal and colon cancer cases separately (as per legend to the right side).

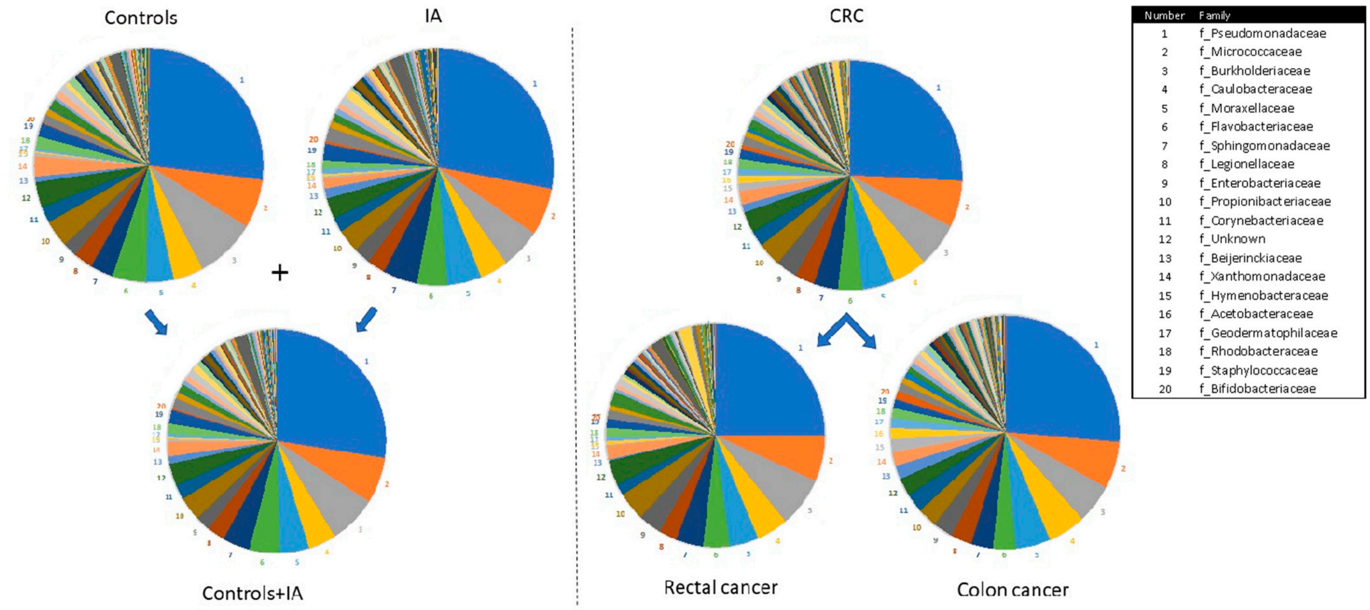

Figure S3. Different taxa between: (A) colorectal cancers (CRC) and controls; (B) CRC and intestinal adenoma (IA); (C) IA and controls by DESeq2 analyses. The taxonomic lineage of each taxon is shown: p, phylum; c, class; o, order; f, family; g, genus; OTU#, Operational Taxonomic Unit. The first two columns show the logarithmic transformation of normalized base mean value for each group. The "padj" column shows the p-value for heterogeneity between groups adjusted for multi-testing analyses. Positive fold changes (shown on a green background) designate taxon overrepresentation in the CRC group. Negative fold changes (shown on a yellow background) designate taxon underrepresentation in the CRC group.

A

CRC	Controls	OTU#	Taxonomy	padj	log2FoldChange
			p_Actinobacteria;c_Acidimicrobiia	3E-02	4.92
			p_Actinobacteria;c_Actinobacteria;o_Bifidobacteriales;f_Bifidobacteriaceae	6E-09	10.21
			p_Actinobacteria;c_Actinobacteria;o_Bifidobacteriales;f_Bifidobacteriaceae;g_Bifidobacterium	6E-09	10.21
			p_Actinobacteria;c_Actinobacteria;o_Corynebacteriales;f_Corynebacteriaceae;g_Corynebacterium	9E-05	-7.55
		OTU_54	p_Actinobacteria;c_Actinobacteria;o_Corynebacteriales;f_Corynebacteriaceae;g_Corynebacterium;s_Corynebacterium£sp.	2E-04	-7.14
			p_Actinobacteria;c_Actinobacteria;o_Frankiales;f_Geodermatophilaceae	2E-02	4.32
			p_Actinobacteria;c_Actinobacteria;o_Micrococcales;f_Micrococcaceae;g_Rothia	8E-03	4.45
			p_Actinobacteria;c_Actinobacteria;o_o_Bifidobacteriales	6E-09	10.21
			p_Actinobacteria;c_Actinobacteria;o_Propionibacteriales;f_Nocardioidaceae	2E-02	-4.17
			p_Actinobacteria;c_Thermoleophilia	5E-06	7.01
			p_Bacteroidetes;c_Bacteroidia;o_Bacteroidales;f_Bacteroidaceae	2E-09	-6.77
			p_Bacteroidetes;c_Bacteroidia;o_Bacteroidales;f_Bacteroidaceae;g_Bacteroides	2E-09	-6.77
			p_Bacteroidetes;c_Bacteroidia;o_Sphingobacteriales	9E-06	-6.42
			p_Bacteroidetes;c_Bacteroidia;o_Sphingobacteriales;f_Sphingobacteriaceaa	1E-04	-5.39
		OTU_62	p_Firmicutes;c_Clostridia;o_Clostridiales;f_Clostridiaceae£1;g_Clostridium£sensu£stricto£1;s_Multi-affiliation	9E-05	-5.04
			p_Firmicutes;c_Clostridia;o_Clostridiales;f_Peptostreptococcaceae	3E-02	4.25
		OTU_49	p_Firmicutes;c_Clostridia;o_Clostridiales;f_Peptostreptococcaceae;g_Romboutsia;s_metagenome	1E-06	9.2
			p_Proteobacteria;c_Alphaproteobacteria;o_Acetobacterales	5E-06	7.38
			p_Proteobacteria;c_Alphaproteobacteria;o_Acetobacterales;f_Acetobacteraceae	5E-06	7.38
		OTU_44	p_Proteobacteria;c_Alphaproteobacteria;o_Caulobacterales;f_Caulobacteraceae;g_Caulobacter;s_Unknown	1E-02	-5.53
			p_Proteobacteria;c_Alphaproteobacteria;o_Sphingomonadales;f_Sphingomonadaceae;g_Novosphingobium	2E-02	4.43
		OTU_42	p_Proteobacteria;c_Alphaproteobacteria;o_Sphingomonadales;f_Sphingomonadaceae;g_Sphingomonas;s_Multi-affiliation	9E-05	-5.34
			p_Proteobacteria;c_Deltaproteobacteria;o_Myxococcales	2E-03	4.46
			p_Proteobacteria;c_Deltaproteobacteria;o_Oligoflexales;f_Oligoflexaceae	9E-05	7.86
		OTU_37	p_Proteobacteria;c_Gammaproteobacteria;o_Betaproteobacteriales;f_Burkholderiaceae;g_Cupriavidus;s_Multi-affiliation	2E-07	-7.02
			p_Proteobacteria;c_Gammaproteobacteria;o_Betaproteobacteriales;f_Burkholderiaceae;g_Ralstonia	4E-02	-4.71
			p_Proteobacteria;c_Gammaproteobacteria;o_Betaproteobacteriales;f_Neisseriaceae	5E-06	6.58
			p_Proteobacteria;c_Gammaproteobacteria;o_Betaproteobacteriales;f_Neisseriaceae;g_Neisseria	2E-02	4.12
			p_Proteobacteria;c_Gammaproteobacteria;o_Enterobacteriales;f_Enterobacteriaceae;g_Pantoea	2E-04	7.73
		OTU_48	p_Proteobacteria;c_Gammaproteobacteria;o_Legionellales;f_Legionellaceae;g_Legionella;s_Unknown	3E-03	4.86
		OTU_83	p_Proteobacteria;c_Gammaproteobacteria;o_Legionellales;f_Legionellaceae;g_Legionella;s_Unknown	3E-03	5.35
		OTU_46	p_Proteobacteria;c_Gammaproteobacteria;o_Pseudomonadales;f_Moraxellaceae;g_Acinetobacter;s_Multi-affiliation	2E-13	10.64
		OTU_31	p_Proteobacteria;c_Gammaproteobacteria;o_Pseudomonadales;f_Moraxellaceae;g_Acinetobacter;s_Multi-affiliation	2E-07	-7.5
		OTU_30	p_Proteobacteria;c_Gammaproteobacteria;o_Pseudomonadales;f_Moraxellaceae;g_Acinetobacter;s_Multi-affiliation	2E-02	4.13

B

IA	CRC	OTU#	Taxonomy	padj	log2FoldChange
		OTU_43	p_Actinobacteria;c_Actinobacteria;o_Corynebacteriales;f_Corynebacteriaceae;g_Corynebacterium£1;s_Unknown	9E-07	7.82
		OTU_94	p_Actinobacteria;c_Actinobacteria;o_Micrococcales;f_Microbacteriaceae;g_Microbacterium;s_Microbacterium£sp.	3E-03	5.23
			p_Actinobacteria;c_Actinobacteria;o_Propionibacteriales;f_f_Nocardioidaceae	2E-03	-4.68
		OTU_6	p_Actinobacteria;c_Actinobacteria;o_Propionibacteriales;f_Propionibacteriaceae;g_Cutibacterium;s_Multi-affiliation	7E-03	1.16
			p_Bacteroidetes;c_Bacteroidia;o_Bacteroidales;f_Bacteroidaceae	1E-05	-4.62
			p_Bacteroidetes;c_Bacteroidia;o_Bacteroidales;f_Bacteroidaceae;g_Bacteroides	1E-05	-4.62
		OTU_52	p_Bacteroidetes;c_Bacteroidia;o_Bacteroidales;f_Bacteroidaceae;g_Bacteroides;s_Multi-affiliation	3E-06	6.65
			p_Bacteroidetes;c_Bacteroidia;o_Chitinophagales	1E-06	7.47
			p_Bacteroidetes;c_Bacteroidia;o_Chitinophagales;f_Chitinophagaceae	3E-04	5.57
			p_Bacteroidetes;c_Bacteroidia;o_Cytophagales;f_f_Hymenobacteraceae	2E-05	5.85
			p_Bacteroidetes;c_Bacteroidia;o_Cytophagales;f_Hymenobacteraceae;g_Hymenobacter	5E-04	4.86
			p_Bacteroidetes;c_Bacteroidia;o_Flavobacteriales;f_Weeksellaceae;g_Cloacibacterium	4E-05	-5.64
		OTU_26	p_Bacteroidetes;c_Bacteroidia;o_Flavobacteriales;f_Weeksellaceae;g_Cloacibacterium;s_Multi-affiliation	7E-05	5.24
			p_Bacteroidetes;c_Bacteroidia;o_Sphingobacteriales	4E-02	-3.75
			p_Bacteroidetes;c_Bacteroidia;o_Sphingobacteriales;f_Sphingobacteriaceae	4E-02	-3.75
			p_Dependentiae	3E-02	-4.12
			p_Dependentiae;c_Babeliae	3E-02	-4.12
			p_Dependentiae;c_Babeliae;o_Babeliales	3E-02	-4.12
			p_Firmicutes;c_Bacilli;o_Bacillales;f_FamilyXI	9E-07	6.9
			p_Firmicutes;c_Bacilli;o_Lactobacillales;f_Lactobacillaceae	2E-07	7.56
			p_Firmicutes;c_Bacilli;o_Lactobacillales;f_Lactobacillaceae;g_Lactobacillus	1E-07	7.7
			p_Firmicutes;c_Clostridia;o_Clostridiales;f_Clostridiaceae;g_Clostridium£sensu£stricto1	3E-09	7.93
			p_Firmicutes;c_Clostridia;o_Clostridiales;f_Lachnospiraceae	4E-02	-4.01
			p_Firmicutes;c_Clostridia;o_Clostridiales;f_Peptostreptococcaceae	5E-12	9.59
			p_Firmicutes;c_Clostridia;o_Clostridiales;f_Peptostreptococcaceae;g_Romboutsia	7E-04	5.89
		OTU_49	p_Firmicutes;c_Clostridia;o_Clostridiales;f_Peptostreptococcaceae;g_Romboutsia;s_metagenome	7E-07	8.36
			p_Firmicutes;c_Clostridia;o_Clostridiales;f_Ruminococcaceae	2E-03	-5.41
			p_Patescibacteria	2E-03	4.76
			p_Patescibacteria;c_Saccharimonadia	8E-07	8.13
			p_Patescibacteria;c_Saccharimonadia,o_Saccharimonadales	8E-07	8.13
			p_Proteobacteria;c_Alphaproteobacteria;o_Acetobacterales	4E-03	4.7
			p_Proteobacteria;c_Alphaproteobacteria;o_Acetobacterales;f_Acetobacteraceae	4E-03	4.7
		OTU_5	p_Proteobacteria;c_Alphaproteobacteria;o_Caulobacterales;f_Caulobacteraceae;g_Unknown;s_Unknown	2E-02	1.27
		OTU_14	p_Proteobacteria;c_Alphaproteobacteria;o_Rhizobiales;f_Rhizobiaceae;g_Phyllobacterium;s_Multi-affiliation	3E-02	-1.71
		OTU_33	p_Proteobacteria;c_Alphaproteobacteria;o_Rhodobacterales;f_Rhodobacteraceae;g_Paracoccus;s_Multi-affiliation	1E-06	6.28
		OTU_42	p_Proteobacteria;c_Alphaproteobacteria;o_Sphingomonadales;f_Sphingomonadaceae;g_Sphingomonas;s_Multi-affiliation	9E-04	-5.01
		OTU_37	p_Proteobacteria;c_Gammaproteobacteria;o_Betaproteobacteriales;f_Burkholderiaceae;g_Cupriavidus;s_Multi-affiliation	4E-05	5.77
			p_Proteobacteria;c_Gammaproteobacteria;o_Betaproteobacteriales;f_Burkholderiaceae;g_Polynucleobacter	2E-02	-6
		OTU_51	p_Proteobacteria;c_Gammaproteobacteria;o_Betaproteobacteriales;f_Burkholderiaceae;g_Ralstonia;s_Multi-affiliation	8E-03	4.77
			p_Proteobacteria;c_Gammaproteobacteria;o_Betaproteobacteriales;f_Neisseriaceae;g_Neisseria	1E-02	4.42
			p_Proteobacteria;c_Gammaproteobacteria;o_Enterobacteriales;f_Enterobacteriaceae;g_Pantoea	4E-05	8.05
		OTU_32	p_Proteobacteria;c_Gammaproteobacteria;o_Legionellales;f_Legionellaceae;g_Legionella;s_Multi-affiliation	1E-04	5.74
		OTU_46	p_Proteobacteria;c_Gammaproteobacteria;o_Pseudomonadales;f_Moraxellaceae;g_Acinetobacter;s_Multi-affiliation	2E-10	8.85
		OTU_31	p_Proteobacteria;c_Gammaproteobacteria;o_Pseudomonadales;f_Moraxellaceae;g_Acinetobacter;s_Multi-affiliation	3E-05	-6.4
		OTU_30	p_Proteobacteria;c_Gammaproteobacteria;o_Pseudomonadales;f_Moraxellaceae;g_Acinetobacter;s_Multi-affiliation	5E-02	3.29
		OTU_18	p_Proteobacteria;c_Gammaproteobacteria;o_Xanthomonadales;f_Xanthomonadaceae;g_Stenotrophomonas;s_Multi-affiliation	5E-10	4.63

C

IA	Controls	OTU#	Taxonomy	padj	log2FoldChange
			p_Actinobacteria;c_Actinobacteria;o_Bifidobacteriales	1E-06	9.25
			p_Actinobacteria;c_Actinobacteria;o_Bifidobacteriales;f_Bifidobacteriaceae	1E-06	9.25
			p_Actinobacteria;c_Actinobacteria;o_Corynebacteriales;f_Corynebacteriaceae;g_Corynebacterium	3E-05	-8.05
		OTU_54	p_Actinobacteria;c_Actinobacteria;o_Corynebacteriales;f_Corynebacteriaceae;g_Corynebacterium;s_Corynebacterium£sp.	3E-04	-7.51
		OTU_43	p_Actinobacteria;c_Actinobacteria;o_Corynebacteriales;f_Corynebacteriaceae;g_Corynebacterium£1;s_Unknown	6E-05	-6.48
			p_Actinobacteria;c_Thermoleophilia	2E-04	7.78
			p_Bacteroidetes;c_Bacteroidia;o_Chitinophagales	3E-09	-8.68
			p_Bacteroidetes;c_Bacteroidia;o_Chitinophagales;f_Chitinophagaceae	6E-09	-8.83
			p_Bacteroidetes;c_Bacteroidia;o_Cytophagales;f_Hymenobacteraceae	4E-03	-4.84
			p_Bacteroidetes;c_Bacteroidia;o_Cytophagales;f_Hymenobacteraceae;g_Hymenobacter	4E-03	-4.85
			p_Bacteroidetes;c_Bacteroidia;o_Cytophagales;f_Spirosomaceae	4E-03	-5.27
			p_Firmicutes;c_Bacilli;o_Lactobacillales;f_Lactobacillaceae	1E-05	-6.85
			p_Firmicutes;c_Bacilli;o_Lactobacillales;f_Lactobacillaceae;g_Lactobacillus	8E-06	-6.99
			p_Firmicutes;c_Clostridia;o_Clostridiales;f_Clostridiaceae;g_Clostridium£sensu£stricto1	5E-13	-9.39
			p_Firmicutes;c_Clostridia;o_Clostridiales;f_Clostridiaceae£1	2E-02	-3.74
		OTU_62	p_Firmicutes;c_Clostridia;o_Clostridiales;f_Clostridiaceae£1;g_Clostridium£sensu£stricto£1;s_Multi-affiliation	6E-05	-5.54
			p_Firmicutes;c_Clostridia;o_Clostridiales;f_Family£XI	8E-10	-9.12
			p_Patescibacteria	3E-02	-3.9
			p_Patescibacteria;c_Saccharimonadia	2E-05	-7.75
			p_Patescibacteria;c_Saccharimonadia;o_Saccharimonadales	2E-05	-7.75
			p_Proteobacteria;c_Alphaproteobacteria;o_Rhizobiales;f_Beijerinckiaceae;g_Bosea	3E-02	-5.05
		OTU_33	p_Proteobacteria;c_Alphaproteobacteria;o_Rhodobacterales;f_Rhodobacteraceae;g_Paracoccus;s_Multi-affiliation	8E-06	-5.41
		OTU_37	p_Proteobacteria;c_Gammaproteobacteria;o_Betaproteobacteriales;f_Burkholderiaceae;g_Cupriavidus;s_Multi-affiliation	3E-10	-8.31
		OTU_71	p_Proteobacteria;c_Gammaproteobacteria;o_Betaproteobacteriales;f_Burkholderiaceae;g_Massilia;s_Massilia£sp.	1E-06	8.81
			p_Proteobacteria;c_Gammaproteobacteria;o_Betaproteobacteriales;f_Neisseriaceae	5E-07	7.14
		OTU_45	p_Proteobacteria;c_Gammaproteobacteria;o_Diplorickettsiales;f_Diplorickettsiaceae;g_Unknown;s_Unknown	3E-02	4.29
		OTU_83	p_Proteobacteria;c_Gammaproteobacteria;o_Legionellales;f_Legionellaceae;g_Legionella;s_Unknown	2E-02	4.36
		OTU_65	p_Proteobacteria;c_Gammaproteobacteria;o_Pseudomonadales;f_Moraxellaceae;g_Acinetobacter;s_Unknown	3E-08	10
		OTU_1	p_Proteobacteria;c_Gammaproteobacteria;o_Pseudomonadales;f_Pseudomonadaceae;g_Pseudomonas;s_Multi-affiliation	6E-03	0.44
		OTU_18	p_Proteobacteria;c_Gammaproteobacteria;o_Xanthomonadales;f_Xanthomonadaceae;g_Stenotrophomonas;s_Multi-affiliation	3E-10	-4.65

Figure S4. Multidimensional scaling (MDS) differentiates bacterial patterns of study blood samples and DNA extraction negative controls on Bray-Curtis distance matrices for all 10 randomized comparisons. Study blood samples (in Red) separated from negative controls (in Blue) in all of the randomized comparisons.

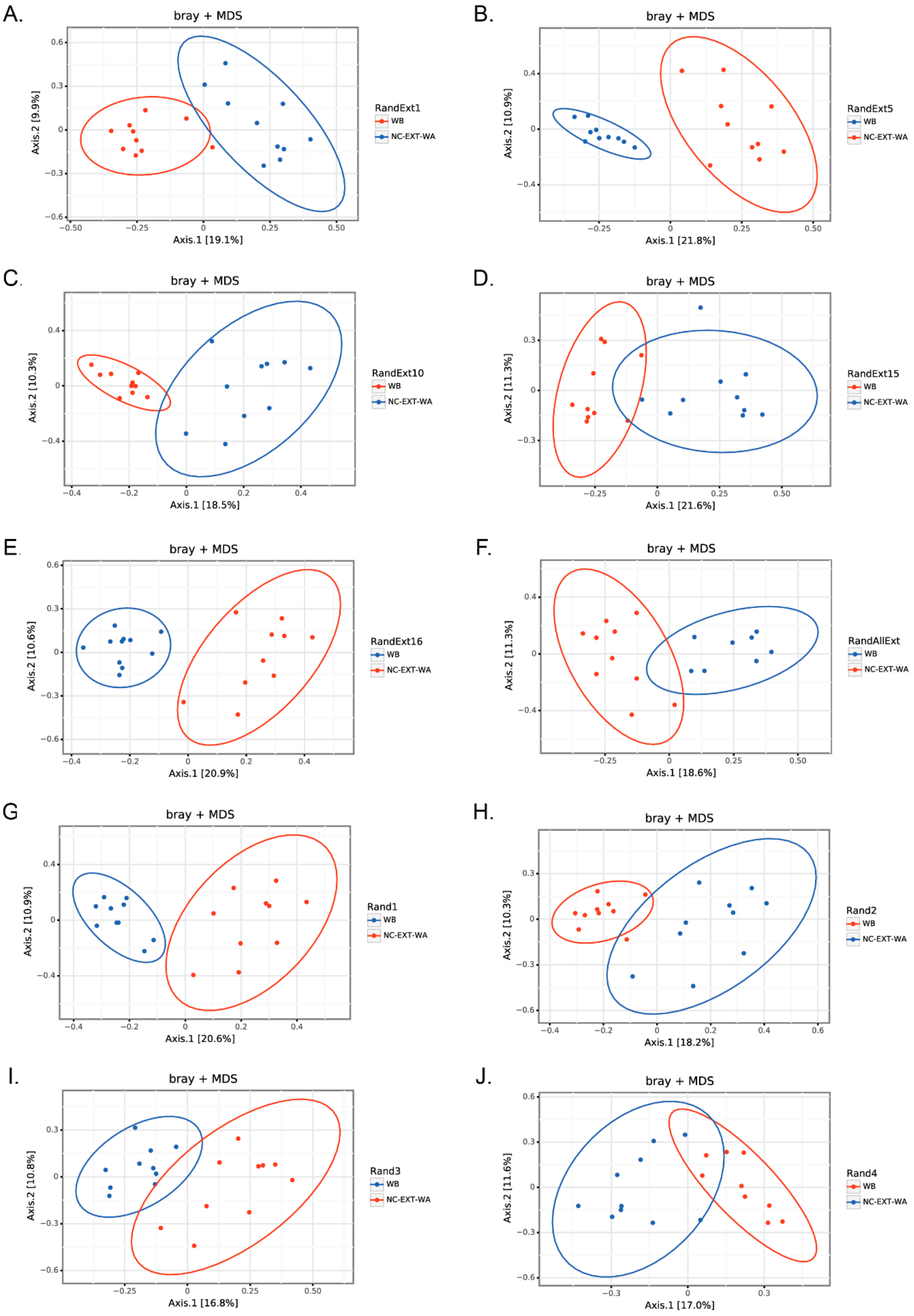

Supplement: Supplementary file 1 [file cancers-13-06363-s001.zip › cancers-1475939-supplementary.pdf]
